# Species identification of crested gibbons (*Nomascus*) in captivity in China using karyotyping- and PCR-based approaches

**DOI:** 10.24272/j.issn.2095-8137.2018.036

**Published:** 2018-04-03

**Authors:** Wen-Hui Nie, Jin-Huan Wang, Wei-Ting Su, Yu Hu, Shui-Wang He, Xue-Long Jiang, Kai He

**Affiliations:** 1 Kunming Institute of Zoology, Chinese Academy of Sciences, Kunming Yunnan 650223, China; 2 Kyoto University Museum, Kyoto University, Kyoto 606-8417, Japan

**Keywords:** F2 hybrid gibbon, Fluorescence *in situ* hybridization, *Nomascus*, Pericentric inversion, Species identification, Animal welfare

## Abstract

Gibbons and siamangs (Hylobatidae) are well-known for their rapid chromosomal evolution, which has resulted in high speciation rate within the family. On the other hand, distinct karyotypes do not prevent speciation, allowing interbreeding between individuals in captivity, and the unwanted hybrids are ethically problematic as all gibbon species are endangered or critically endangered. Thus, accurate species identification is crucial for captive breeding, particularly in China where studbooks are unavailable. Identification based on external morphology is difficult, especially for hybrids, because species are usually similar in appearance. In this study, we employed G-banding karyotyping and fluorescence *in situ* hybridization (FISH) as well as a PCR-based approach to examine karyotypic characteristics and identify crested gibbons of the genus *Nomascus* from zoos and nature reserves in China. We characterized and identified five karyotypes from 21 individuals of *Nomascus*. Using karyotypes and mitochondrial and nuclear genes, we identified three purebred species and three hybrids, including one F2 hybrid between *N. gabriellae* and *N. siki*. Our results also supported that *N. leucogenys* and *N. siki* shared the same inversion on chromosome 7, which resolves arguments from previous studies. Our results demonstrated that both karyotyping and DNA-based approaches were suitable for identifying purebred species, though neither was ideal for hybrid identification. The advantages and disadvantages of both approaches are discussed. Our results further highlight the importance of animal ethics and welfare, which are critical for endangered species in captivity.

## INTRODUCTION

The rates of chromosomal evolution are an order of magnitude higher in Mammalia than in most other classes of vertebrates and are strongly correlated with high speciation rates (Bush et al., 1977). High karyotypic diversity and rapid speciation are often observed in taxa characterized by small effective population sizes, which includes the gibbons and siamangs of the small ape family Hylobatidae (Primate). These animals are endemic to southern China, South Asia, and Southeast Asia, and have been assigned into four genera (*Hoolock*, *Hylobates*, *Nomascus*, and *Symphalangus*; Brandon-Jones et al., 2004; Roos & Geissmann, 2001). While siamangs (*Symphalangus*) possess a unique and large gular sac (throat pouch), gibbons share very similar external morphology, and were long acknowledged as a single genus (*Hylobates*). However, *Hoolock* and *Nomascus* were subsequently recognized as full genera based on karyotypic studies, which revealed a unique diploid number (2*n*) of chromosomes among each genus: *Hoolock* (2*n*=38), *Hylobates* (2*n*=44), *Nomascus* (2*n*=52), and *Symphalangus* (2*n*=50) (Chiarelli, 1972; Prouty et al., 1983; Van Tuinen & Ledbetter, 1983). This subdivision, as supported by molecular phylogenetic studies, occurred in the Late Miocene approximately 7 Ma (Fan et al., 2017; Springer et al., 2012). Today, 20 species of gibbons and siamangs are recognized, though the number continues to increase due to newly discovered species (Fan et al., 2017).

Speciation in this family is considerably faster than that observed in many other groups of mammals and is considered to be the result of high frequency of chromosomal rearrangement (Misceo et al., 2008), including compound inversion/translocation (Jauch et al., 1992; Koehler et al., 1995a, 1995b; Marks, 1982; Nie et al., 2001; Stanyon & Chiarelli, 1982, 1983; Van Tuinen & Ledbetter, 1983; Yu et al., 1997). Previous comparison between the gibbon (*N. leucogenys*) and human genomes revealed 100 synteny breakpoints, which can facilitate chromosome breakage and rearrangement (Carbone et al., 2006). Gibbons exhibit highly structured society in their populations, which may promote chromosome rearrangement fixation, thereby allowing rapid speciation (Wilson et al., 1975).

The crested gibbon genus *Nomascus* comprises seven recognized species, including *N. concolor* (western black-crested gibbon), *N. gabriellae* (yellow-cheeked gibbon), *N. hainanus* (Hainan gibbon), *N. leucogenys* (northern white-cheeked gibbon), *N. nasutus* (eastern black-crested gibbon), *N. siki* (southern white-cheeked gibbon) (Brandon-Jones et al., 2004; Roos & Geissmann, 2001), and the recently discovered *N. annamensis* (northern buffed-cheeked gibbon) (Thinh et al., 2010). These species are characterized by a variety of morphological, anatomical, karyological, and vocal features (Couturier & Lernould, 1991; Garza & Woodruff, 1994; Schilling, 1984; Thinh et al., 2011).

Although all studied *Nomascus* species have a unique diploid number of 52 (Couturier et al., 1982; Dutrillaux et al., 1975; Van Tuinen & Ledbetter, 1983), they also exhibit considerable interspecific karyotypic variation. Using the R-banding technique, Couturier & Lernould (1991) distinguished the karyotypes for six individuals of *N. leucogenys*, two *N. siki*, three *N. gabriellae*, and several hybrids using a combination of an inversion on chromosome 7 and a translocation between chromosomes 1 and 22. The inversion on chromosome 7 was also confirmed based on the number of hybridization signals provided by human chromosome probes 4 and 22. On unrearranged chromosome 7, one hybridization signal of probe 4 on 7q and one signal of probe 22 on 7p were detected. On rearranged chromosome 7, two signals of each probe were detected, one on each arm of the chromosomes (Koehler et al., 1995b). Heterozygous translocation t (1; 22) has also been identified in hybrids of *N. leucogenys* and *N. gabriellae* (Couturier et al., 1982; Turleau et al., 1983). Using a PCR-based approach, Carbone et al. (2009) detected the inversion on chromosome 7 in *N. leucogenys* but not in *N. siki*. These results disagreed with those of Couturier & Lernould (1991), and thus need to be revisited and clarified using additional samples.

Currently, most gibbon species are endangered or critically endangered. Several species, including *Hylobates lar* and *N. leucogenys*, may have been extirpated from China largely due to poaching. Reintroducing animals from zoos to their original habitat is a goal for conservation but requires accurate identification of purebred animals. Extreme caution must be undertaken in captive breeding as interspecific and intergeneric hybridization can occur between gibbons and siamangs (Hirai et al., 2007; Myers & Shafer, 1979), which do not resulting in reduced fertility among offspring in certain cases (Van Tuinen et al., 1999). Hybrid offspring are difficult to visually identify due to their similar external morphology, with identification using the karyotypic approach considered more reliable. Co-housing of different species has been avoided in many countries and animals are karyotyped for identification, with studbooks maintained as a database for appropriate management. However, this system is not yet well established in China and different species are usually housed together resulting in hybridization and unknown interbreeding history.

In the current study, we examined 21 gibbons using a combination of G-banding karyotyping and fluorescence *in situ* hybridization (FISH). We also sequenced mitochondrial and nuclear fragments across a known inversion breakpoint on chromosome 7 identified in a previous study (Carbone et al., 2009). We examined whether the combination of G-banding and FISH is a reliable approach for species identification. We evaluated the efficiency and accuracy of karyotyping- and PCR-based approaches for the identification of purebred animals and hybrids. We also re-examined whether the pericentric inversion on chromosome 7 is species-specific in *N. leucogenys*.

## MATERIALS AND METHODS

### Samples

We collected peripheral blood from 20 gibbon individuals raised in Nanning Zoo, Ningbo Zoo, Kunming Zoo, and Huanglianshan National Natural Reserve, and obtained one sample (No.14 in Table 1) of cultured gibbon cells maintained at the Kunming Institute of Zoology, Chinese Academy of Sciences. Fourteen individuals were female and seven were male. All experimental procedures and animal care were performed according to the protocols approved by the Ethics Committee of the Kunming Institute of Zoology, Chinese Academy of Sciences.

**Table 1:** Samples used in this study and a summary of karyotypic characteristics including the FISH signals on the chromosome 7, the lengths of chromosomes 1 and 22, the results of PCR using primer sets BP and HSA, as well as the identification results based on each approach

Nick Name	House	FISH (signal on chr. 7) (pairs)	Chr. 1	Chr. 22	Karyotype	BP	HSA	Cyt *b* genes	Final identification
Su-Su	Nanning Zoo	2	Long (Normal)	Short (Normal)	*N. leucogenys*	N/A	N/A	N/A	N/A
Bei-Li	Nanning Zoo	2	Long (Normal)	Short (Normal)	*N. leucogenys*	+	−	*N. leucogenys*	*N. leucogenys*
Gou-Dan	Nanning Zoo	2	Long (Normal)	Short (Normal)	*N. leucogenys*	+	−	*N. leucogenys*	*N. leucogenys*
San-Mei	Nanning Zoo	2	Long (Normal)	Short (Normal)	*N. leucogenys*	N/A	N/A	N/A	N/A
No14	Missing	2	Long (Normal)	Short (Normal)	*N. leucogenys*	+	−	*N*. cf. *leucogenys*	*N*. cf. *leucogenys*
No name	Kunming Zoo	2	Long (Normal)	Short (Normal)	*N. leucogenys*	N/A	N/A	N/A	N/A
NB1	Ningbo Zoo	2	Long (Normal)	Short (Normal)	*N. leucogenys*	N/A	N/A	N/A	N/A
NB2	Ningbo Zoo	2	Long (Normal)	Short (Normal)	*N. leucogenys*	N/A	N/A	N/A	N/A
HLS3	Huanglianshan Nature Reserve	2	Long (Normal)	Short (Normal)	*N. leucogenys*	N/A	N/A	N/A	N/A
HLS4	Huanglianshan Nature Reserve	2	Long (Normal)	Short (Normal)	*N. leucogenys*	N/A	N/A	N/A	N/A
Fang-Fang	Nanning Zoo	2	Short	Long	*N. siki*	N/A	N/A	N/A	N/A
E’gui	Nanning Zoo	2	Short	Long	*N. siki*	+	−	*N. siki*	*N. siki*
Qingguangyan	Nanning Zoo	2	Short	Long	*N. siki*	N/A	N/A	N/A	N/A
Laoer	Nanning Zoo	2	Short	Long	*N. siki*	N/A	N/A	N/A	N/A
Da-Shan	Nanning Zoo	2	Short	Long	***N. siki***	+	−	***N. gabriellae***	*N. siki* ♂ × (*N. siki* ♂ × *N. gabriellae* ♀) ♀
316	Nanning Zoo	1	Short	Long	*N. gabriellae*	−	+	*N. gabriellae*	*N. gabriellae*
Bai-Shou	Nanning Zoo	1	Short	Long	*N. gabriellae*	−	+	*N. gabriellae*	*N. gabriellae*
Jing-Jing	Nanning Zoo	1	Short	Long	*N. gabriellae*	−	+	*N. gabriellae*	*N. gabriellae*
Mei-Mei	Nanning Zoo	2	1 short, 1 long	1 short, 1 Long	*N. leucogenys*×*N. siki*	+	−	*N. leucogenys*	*N. leucogenys*×*N. siki*
Xiao-Xiao	Nanning Zoo	1 and a half	Short	Long	*N. gabriellae*×*N. siki*	+	+	*N. leucogenys*	*N. gabriellae*×*N. siki*
A-Huang	Nanning Zoo	1 and a half	Short	Long	*N. gabriellae*×*N. siki*	N/A	N/A	N/A	N/A

The karyotype and mitochondrial of Da-Shan suggest different specific affinities. +: The positive result of PCR using primer set BP or HAS. −: The negative result of PCR using primer set BP or HAS. N/A: Not available.

### Cell culture, metaphase preparation, and G-banding

Chromosome suspensions were obtained from lymphocyte cultures. Cell culture and metaphase preparations were performed following conventional procedures (Hungerford, 1965). Briefly, whole blood was cultivated in the presence of phytohaemagglutinin in RPMI 1640 medium supplemented with 10% fetal bovine serum. After 68–70 h of growth, colchicine was added to the cell cultures to a final concentration of 0.4–0.8 μg/mL. The cell cultures were incubated for another 2–4 h before harvest. We treated the cells with hypotonic solution (0.075 mol/L KCl) for 20 min, with thrice fixation in 3:1 methanol/glacial acetic acid. Slides were prepared by applying 10 μL of metaphase suspension onto dry and clean slides, which were then allowed to air dry. G-banding was performed following Seabright (1971). All karyotypes were analyzed after G-banding.

### FISH, image capture, and processing

We used a biotin-labeled probe of the human 22 chromosome. Fluorescence *in situ* hybridization, detection, image capture, and processing were carried out following Yang et al. (2000) and Nie et al. (2002). We detected fluorescence signals using a layer of Cy3-avidin (1:1 000 dilution; Amersham Pharmacia Biotech, USA). After detection, slides were mounted in Vectashield mounting medium with DAPI (4${}^{\prime }$6-diamidino-2-phenylindole, Vector Laboratories, USA). Digital images were acquired using a CytoVision system (Applied Imaging Co., USA) with a CCD camera mounted on a Zeiss microscope (Germany). We associated the hybridization signals with specific chromosome regions based on DAPI-banding patterns.

### Amplification, sequencing, and species identification

After karyotyping, we extracted total DNA from the cultured cells using a commercial DNA extraction kit (Blood & Cell Culture DNA Mini Kit, Qiagen, Germany). The DNA extracted from 10 samples was considered acceptable for subsequent analyses as other samples were not well preserved after karyotyping. We amplified and sequenced cyt *b* genes using the primer pair L14724_hk3 (5${}^{\prime }$-GGACTTATGACATGAAAAATCATCGTTG-3${}^{\prime }$) and H15915_hk3 (5${}^{\prime }$-GATTCCCCATTTCTGGTTTACAAGAC-3${}^{\prime }$). We also conducted PCR using the primers provided in Carbone et al. (2009) (263C9_BP_L with 263C9_BP_R (BP hereafter) and 263C9_BP_L with 263C9_HSA22_R (HSA hereafter)), targeting a fragment across a breakpoint on chromosome 7. According to Carbone et al. (2009), HSA primers should amplify a fragment across a breakpoint in species without an inversion (e.g., *Hylobates* spp., *N. gabriellae*, and *N. siki*), and BP primers should result in positive amplification only for *N. leucogenys*. The mitochondrial and nuclear amplicons were sequenced using a ABI 3730 sequencer (Applied Biosystems, USA).

We identified species based on the karyotyping and sequencing results using the following strategy: (1) we identified the species based on its karyotype; (2) we verified the karyotyping results using PCR with primer sets BP and HSA; (3) we BLAST each cyt *b* gene against the GenBank nucleotide database; (4) we estimated a gene tree to verify the BLAST results using our cyt *b* genes accompanied by a set of sequences representing the recognized *Nomascus* species downloaded from GenBank; and (5) we repeated the karyotyping and amplification/sequencing in triplicate in cases where the mitochondrial, nuclear sequencing, and karyotyping results conflicted (e.g., sample Da-Shan, see Results and Discussion). A final purebred or hybrid and species identification was determined.

We constructed the maximum likelihood gene tree using RAxML v8.2.10 and the CIPRES Science Gateway (Stamatakis, 2014). In addition to the obtained cyt *b* sequences, 17 cyt *b* sequences representing seven recognized species were downloaded from GenBank (Supplementary Table S1) and aligned with our sequences using MAFFT v7.3 (Katoh & Standley, 2014). We partitioned the alignments by codon positions (three partitions). We used GTR+G as the evolutionary model for each partition as RAxML does not accept models other than GTR or GTR+G. We ran each analysis using the rapid bootstrapping algorithm and let RAxML halt bootstrapping automatically.

## RESULTS

### Identified karyotypic species

**Figure 1:**
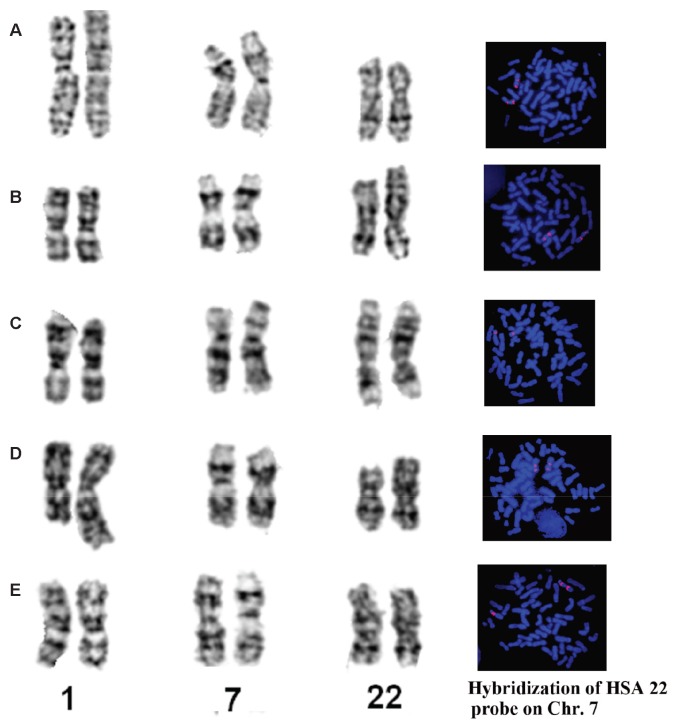
Comparison of G-banded chromosomes 1, 7, and 22 and FISH results with human 22 chromosome-specific probe in different Nomascus species and their hybrids

The diploid number (2*n*) of all analyzed gibbons was 52. G-banding revealed different lengths of chromosomes 1 and 22, and FISH revealed two, three, or four fluorescence signals on chromosome 7 (Figure 1). Considering the G-banded karyotyping and FISH results, we recognized five karyotypes representing three karyotypic species and two hybrids, with the latter two distinguished by nonhomologous pairing.

A: *Nomascus leucogenys*; B: *Nomascus siki*; C: *Nomascus gabriellae*; D: hybrid of *N. leucogenys*×*N. siki*; E: hybrid of *N. gabriellae*×*N. siki*.

#### *Nomascus leucogenys* (Figure 2)

The G-banded karyotypes of 10 individuals (7♀, 3♂) were similar to the G-banded (Van Tuinen & Ledbetter, 1983) and R-banded karyotypes for *N. leucogenys* (Couturier & Lernould, 1991). Chromosomes 1 and 22 were normal. Two pairs of FISH signals were found on chromosome 7, supporting a pericentric inversion event (Figure 1A).

**Figure 2:**
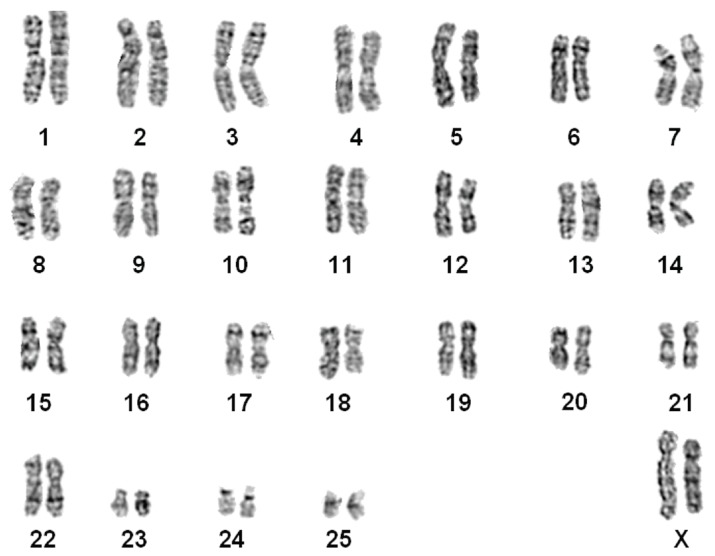
G-banded karyotype of a northern white-cheeked gibbon (*N. leucogenys*)

#### *Nomascus siki* (Figure 3)

The karyotypes of five samples (3♀, 2♂) were identical to those of *N. siki* reported by Couturier & Lernould (1991). Compared with *N. leucogenys*, a balanced translocation between chromosome pairs 1 and 22 was detected, resulting in one pair of shortened chromosome 1 and one pair of derived chromosome 22 (t (1; 22)). Similar to that observed in *N. leucogenys*, FISH demonstrated a pericentric inversion event on chromosome 7 (Figure 1B). The sample “Da-Shan” had a typical siki-karyotype.

**Figure 3:**
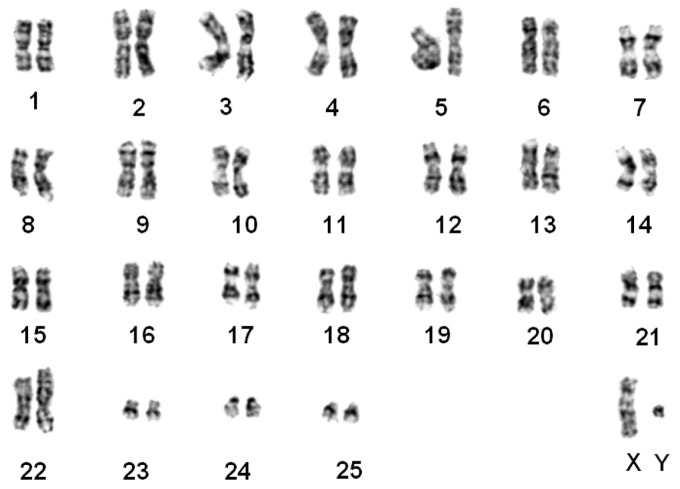
G-banded karyotype of a southern white-cheeked gibbon (*N. siki*)

#### *Nomascus gabriellae* (Figure 4)

The G-banded karyotypes of three animals (1♀, 2♂) were the same as that of *N. gabriellae* (Couturier & Lernould, 1991). This karyotype was similar to *N. siki* in the presence of t (1; 22). Only one pair of FISH signals were observed on chromosome 7, thus differing from *N. siki* and *N. leucogenys* (Figure 1C).

**Figure 4:**
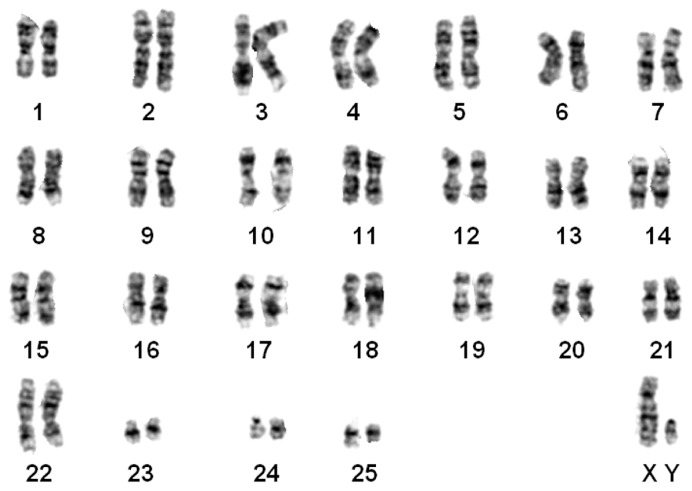
G-banded karyotype of a yellow-cheeked gibbon (*N. gabriellae*)

#### *Nomascus leucogenys*×*Nomascus* siki hybrid (Figure 5)

The G-banded karyotype of one specimen was unique, characterized by a heterozygous translocation. One chromosome 1 and 22 were normal, similar to that of *N. leucogenys*, and the other chromosome 1 and 22 were similar to that of *N. siki*, indicating one translocation t (1; 22). Two pairs of FISH signals were observed on chromosome 7, indicating a pericentric inversion event (Figure 1D). This specimen was identified as a hybrid of *N. leucogenys*×*N. siki*.

**Figure 5:**
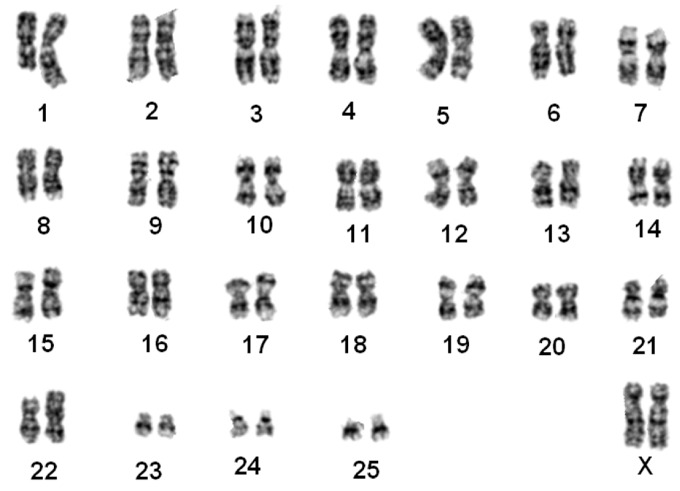
G-banded karyotype of a *N. leucogenys*×*N. siki* hybrid

#### *Nomascus gabriellae*×*Nomascus siki* hybrid (Figure 6)

The distinct G-banded karyotype of two individuals was similar to that of *N. siki* and *N. gabriellae* in the presence of t (1; 22). FISH detected three fluorescence signals on chromosome 7, indicating heterozygous pericentric inversion (Figure 1E). These two individuals were identified as hybrids of *N. gabriellae*×*N. siki*.

**Figure 6:**
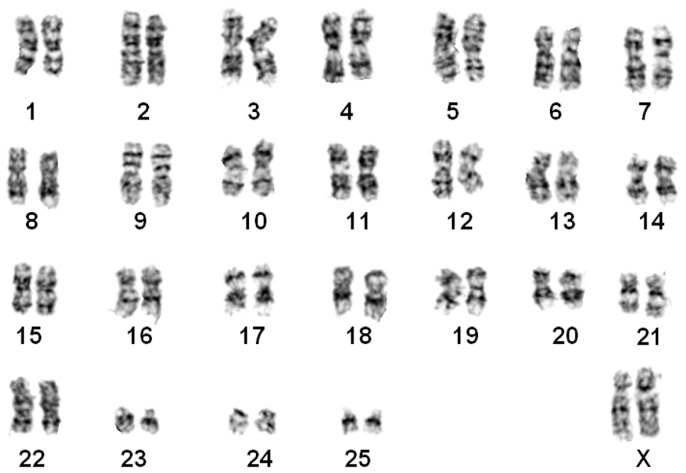
G-banded karyotype of a *N. leucogenys* ×*N. siki* hybrid

### Mitochondrial and nuclear sequences

**Figure 7:**
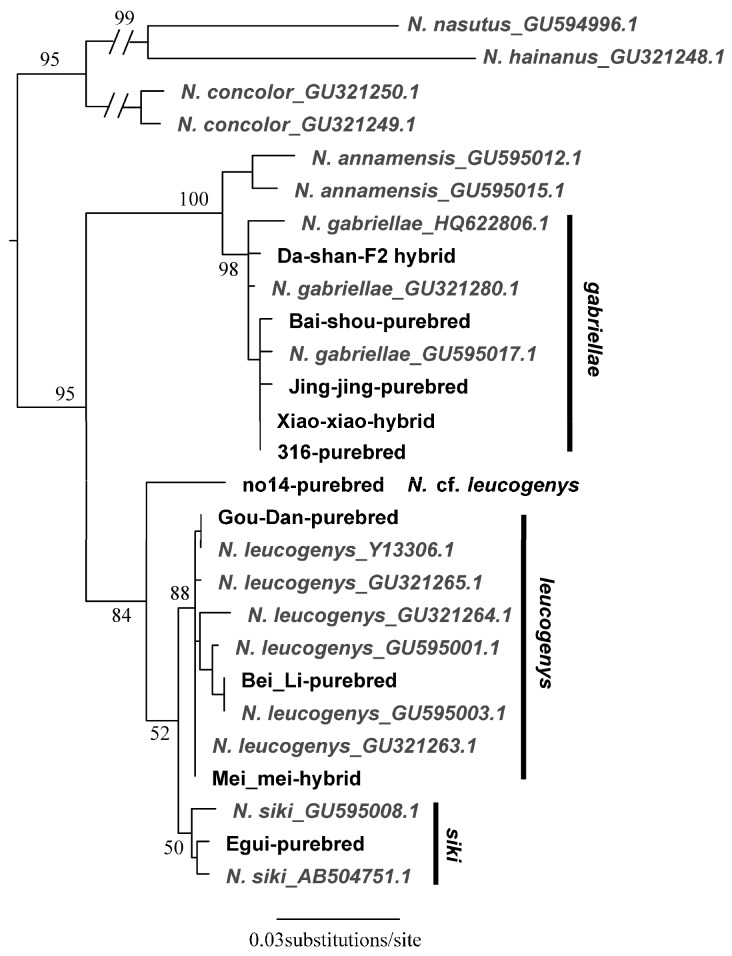
Maximum-likelihood gene tree estimated using the cyt *b* genes of *Nomascus* species

We determined the complete cyt *b* sequences for 10 individuals representing the three purebred species (*n*=7) and two hybrids (*n*=3) identified in the karyotypic analyses. All new sequences are available in GenBank under accession Nos. MH188408–MH188428 (Supplementary Table S1). Five out of the seven purebred animals were supported by BLAST and phylogenetic analyses using the cyt *b* gene and the other two were not (Figure 7). One sample (Da-Shan) was identified as *N. siki* based on its karyotype, but its mitochondrial gene was typical of *N. gabriellae*, which also supported by our phylogenetic analyses (bootstrap value (BS)=98). Sample 14 was identified as *N. leucogenys* based on its karyotype but could not be identified unambiguously as it was equally similar to *N. leucogenys* and *N. siki*. It was closely related to (BS=84) and formed a sister lineage of these two species on the phylogenetic tree (BS=52). The cyt *b* genes of the two hybrids identified in the karyotypic analyses (Xiao-Xiao and Mei-Mei) corresponded to one of their parental species (BS≥88). On our cyt *b* genetic tree (Figure 7), sample 14 was a close relative to (BS=84) and formed a sister lineage with *N. siki*+*N. leucogenys* (BS=52).

Branch lengths correspond to substitution rates. Numbers on branches are bootstrap values. Sequences downloaded from GenBank are in gray and GenBank accession Nos. are shown.

The PCR results using the HSA and BP primer sets were concordant with the karyotypic results. The PCR results using the BP primers (confirming the inversion) were positive for purebred *N. leucogenys*, *N. siki*, and the *N. leucogenys*×*N. siki* hybrid (Mei-Mei) and were negative when using the HSA primers for these samples (Table 1). The PCR results using the BP primers were negative for purebred *N. gabriellae* but positive when using the HSA primers. For the hybrid identified as *N. gabriellae*×*N. siki* (Xiao-Xiao), PCR and sequencing were successful using both BP and HSA primers. For Da-Shan, the PCR results were positive using BP primers and negative using HSA primers, which were congruent with its karyotype (*siki* type) but disagreed with its mitochondrial gene results.

Because the karyotype and PCR results were congruent, we assigned seven samples as purebred species and identified another two as hybrids. One sample (14) was identified as *N*. cf. *leucogenys* as it did not cluster with the other *N. leucogenys* sequences downloaded from GenBank. We considered Da-Shan as an F2 hybrid of *N. gabriellae* and *N. siki* due to the inconsistent patterns revealed by karyotyping and PCR using the HSA and BP primers (*siki*), and the mitochondrial phylogeny (*gabriellae*) (Table 1).

## DISCUSSION

We used karyotypic- and PCR-based approaches to examine the affinities of *Nomascus* species characterized by high karyotypic diversity. We determined the specific karyotypes of each species as well as the existence of an inversion on chromosome 7 in *N. siki* and *N. leucogenys*. We also revealed the definitive existence of hybrids in zoos in China, which calls attention to the animal ethics and welfare issues related to endangered species breeding in captivity. Using the *Nomascus* species as our focal taxa, we evaluated the accuracy and efficiency of both approaches in identifying purebred and hybrid species.

Our results supported that *N. gabriellae*, *N. leucogenys*, and *N. siki* are distinguishable based on translocation t (1; 22) and inversion on chromosome 7 (Figure 1, Figure 2, Figure 3, Figure 4 and Figure 5), even though *N. leucogenys* and *N. siki* diverged from each other only recently. Couturier & Lernould (1991) revealed that *N. hainanus* does not have an inversion on chromosome 7 or translocation t (1; 22), and therefore has a distinct karyotype. It would be interesting to examine the G-banded karyotypes and FISH results of *N. concolor* as well as *N. annamensis*, which is sister to *N. gabriellae* and recognized only recently (Thinh et al., 2010). Due to interspecific karyotypic variation, hybrids can be easily distinguished based on heterozygous R-, G-banded karyotypes and FISH results (Couturier & Lernould, 1991; this study). In wild populations, hybridization of *Hylobates* species such as *H. lar*×*H. pileatus*, *H. lar*×*H. agilis*, and *H. muelleri*×*H. agilis*/*H. albibarbis* has been observed (Brockelman & Gittins, 1984). Hybridization between *Nomascus* species in the wild has not been reported, which is likely due to their allopatric distribution. However, we observed heterozygous karyotypes in captive *Nomascus* animals, congruent with previous study (Couturier & Lernould, 1991). Our results support the possibility of nonhomologous pairing during meiophase (Figure 5, Figure 6), which is an interesting characteristic in Hylobatidae. Because gibbons have very strong social structures and usually live in small family groups, such nonhomologous pairing may be easily fixed during evolutionary histories and further promote diversification and speciation, resulting in high species diversity within the family.

Our FISH and DNA analyses supported the existence of an inversion on chromosome 7 in both *N. leucogenys* and *N. siki* (Figure 1, Figure 2 and Figure 3), consistent with the findings of Couturier & Lernould (1991), though not supported by Carbone et al. (2009). The negative results obtained in the latter study may be due to the hair sample used to represent *N. siki*, which is known for low DNA quantity as well as the existence of PCR inhibitors.

The phylogenetic position of sample 14 was an interesting finding. Its karyotype was identical to the other *N. leucogenys* samples (Table 1), which was not supported by the mitochondrial gene tree (Figure 7). We repeated amplification and sequencing three times for this sample. The sequences were identical, indicating no cross-sample contamination, and no premature codon was observed, indicating it was not a pseudogene. There are two hypothetical scenarios that may explain these results. One is incomplete lineage sorting between *N. leucogenys* and *N. siki*, resulting in non-monophyletic relationships. This situation has never been observed because the effective population sizes of gibbon species are usually small. Alternatively, this sample might represent a distinct and unknown taxon, which is a close relative but not identical to *N. leucogenys* or *N. siki*. The sample was obtained from the Kunming Institute of Zoology, Chinese Academy of Sciences, in 1993 and the information on the animal was not recorded in detail. Unfortunately, neither hypothesis could be supported or rejected in the current study.

Similar to previous studies, our study supported that hybridization has occurred in captivity. However, fertility may be less impacted as identification of Da-Shan showed it to be a likely F2 hybrid of *N. gabriellae* and *N. siki*, despite there being no available studbook. This is the first record of a cross-back F2 hybrid in *Nomascus* as most other countries have well-established systems in place to prevent interbreeding of gibbons in captivity, with many animals also previously karyotyped. Continuous interbreeding and production of fertile offspring may spread across zoos in China because there are only limited numbers of gibbons in zoos, and correct species identification of their offspring will become far more difficult after several generations and recombination. Our findings call for the introduction of a system to prevent gibbon interbreeding in captivity and for better welfare and awareness of these animals.

The R-banding technique can distinguish different karyotypes of *Nomascus* species (Couturier & Lernould, 1991), but is both difficult and time-consuming. Herein, we demonstrated that G-banding in combination with FISH is a reliable approach for identifying karyotypic characteristics. This approach was also appropriate for species identification, though it was limited in identifying cross-back hybrid F2 individuals and did not recognize Da-Shan as a hybrid (Table 1). As per Carbone et al. (2009), we agree that PCR and sequencing can be applicable for examining chromosome inversion and species identification. This approach does not require high quality cultured cells or karyotyping techniques and is applicable for DNA samples with low quality and/or low yield, which certainly include, but are not limited to saliva, urine, hair, and feces. The known limitation is that accurate identification of hybrids relies on a finely established system with known karyotypes, breakpoints, and primers. In our case, it easily distinguished *N. leucogenys*/*N. siki* from *N. gabriellae*, but could not easily distinguish *N. leucogenys*/*N. siki* from the hybrid of *N. leucogenys*×*N. siki*.

## Supplementary Material

http://www.zoores.ac.cn/fileup/2095-8137/SUPPL/20180720193024.zip
